# External validation and cross-center transportability of phenotypic age acceleration and laboratory frailty for postoperative mortality prediction: a dual-center cohort study

**DOI:** 10.3389/fendo.2026.1970011

**Published:** 2026-10-27

**Authors:** Rundong Tang, Jingyi Yu, Xiang Ji

**Affiliations:** 1 Department of Anesthesiology, The Affiliated Hospital of Qingdao University, Qingdao, Shandong, China; 2 The Second Affiliated Hospital, Shandong First Medical University and Shandong Academy of Medical Sciences, Taian, China; 3 The Affiliated Taizhou People’s Hospital of Nanjing Medical University, Taizhou, China; 4 Department of Anesthesiology, Binhai County People’s Hospital, Yancheng, Jiangsu, China

**Keywords:** biological aging, external validation, frailty, laboratory frailty index, phenotypic age acceleration, postoperative mortality, transportability

## Abstract

**Background:**

Phenotypic Age Acceleration (PAA) and the Laboratory Frailty Index (FI-Lab) can be derived from routine biomarkers to describe biologic aging and accumulated laboratory deficits. Their prognostic behavior across different clinical settings, however, remains uncertain. We examined whether associations with postoperative mortality were consistent between two hospitals and whether either measure added predictive information beyond standard clinical variables.

**Methods:**

We analyzed 1,223 adults (671 from Shandong and 552 from Jiangsu) with linked health-examination/questionnaire information, perioperative characteristics, and follow-up through 540 days. Phenotypic Age was calculated from chronological age and nine biomarkers. Primary PAA used the Shandong age–Phenotypic Age relation as a fixed reference, and FI-Lab was the proportion of 17 prespecified laboratory deficits. Center-specific and center-stratified Cox models assessed 540-day mortality. Ridge logistic models were trained in Shandong and externally validated in Jiangsu for 365-day mortality; the reverse direction was treated as a secondary reciprocal transportability sensitivity analysis. As the fixed Shandong reference induced age dependence in Jiangsu, sensitivity analyses recalculated PAA as center-specific age–Phenotypic Age residuals with center-specific standardization. Secondary analyses covered the 12-month composite of death or incident diabetes, chronic kidney disease, or cardiovascular disease. Longitudinal analyses of within-person PAA and FI-Lab changes were exploratory; exact person-specific reassessment intervals were unavailable, and follow-up chronological age for PAA was approximated as baseline age + 1 year. All laboratory measurements used to calculate PAA and FI-Lab were obtained from the preoperative panel within 24 h before the index operation, and completeness of this panel was required for inclusion.

**Results:**

During 1,694.66 person-years, 74 participants died, including 57 within 365 days. In the fully adjusted center-stratified model, each 1-SD increase in PAA was associated with mortality [hazard ratio (HR), 1.34; 95% confidence interval (CI), 1.03–1.75], whereas the FI-Lab estimate was borderline (HR, 1.28; 95% CI, 1.00–1.65). Cross-center interaction estimates were imprecise (PAA interaction ratio, 1.06; 95% CI, 0.68–1.65; FI-Lab interaction ratio, 1.48; 95% CI, 0.94–2.32). Fixed-reference PAA was strongly inversely correlated with chronological age in Jiangsu (*r* = − 0.74); when PAA was recalculated using center-specific age–Phenotypic Age residuals and standardized within center, the fully adjusted pooled association remained (HR, 1.39; 95% CI, 1.09–1.77). In the primary Shandong-to-Jiangsu external validation, the study clinical model had an area under the receiver operating characteristic curve (AUC) of 0.60 (95% CI, 0.45–0.73); adding PAA, FI-Lab, or both yielded AUCs of 0.60, 0.63, and 0.63, respectively, without a consistent decision-curve net-benefit gain. FI-Lab, but not PAA, was associated with the 12-month death-or-incident-disease composite (RR, 1.36; 95% CI, 1.08–1.71). Among 820 participants with repeated measurements, exploratory mean changes were + 2.90 years for PAA and + 0.02 for FI-Lab. Calibration estimates in the primary external-validation cohort were imprecise because only 23 deaths occurred by 365 days; nonsignificant calibration tests were not interpreted as evidence of good calibration.

**Conclusions:**

Laboratory-derived aging and frailty measures were related to postoperative mortality across the two cohorts, and the PAA association was robust to center-specific residualization. However, absolute fixed-reference PAA values were not directly transportable across centers because the age–Phenotypic Age relations differed. Neither marker consistently improved prediction beyond the study clinical model, whose external discrimination was modest; calibration remained imprecise. Larger multicenter studies with rigorous cross-center laboratory harmonization are needed before routine perioperative use.

## 1 Introduction

Perioperative risk assessment commonly considers chronological age, comorbidity, functional status, and surgical stress. Even with similar profiles on these measures, patients can differ substantially in their tolerance for surgery. Frailty captures part of this variation and is associated with adverse surgical outcomes (1, 2). A meta-analysis likewise found higher postoperative morbidity and mortality among frail patients (3), and longer-term associations have been reported after cardiac and colorectal surgery (4, 5). Measures of physiologic reserve may therefore add information that chronological age alone does not provide.

Frailty can be measured in several ways, each with different data requirements and practical advantages. The Fatigue, Resistance, Ambulation, Illnesses, and Loss of weight (FRAIL) questionnaire is brief and readily used in large cohorts (6), whereas physical-phenotype and deficit-accumulation approaches cover broader domains of health (7, 8). As these instruments capture related but nonidentical constructs, consensus guidance does not favor a single tool for every setting (9). Laboratory-based measures offer another option: they use tests already obtained in routine care and can capture physiologic abnormalities without additional performance testing.

Phenotypic Age is a mortality-derived measure of biologic age calculated from chronological age and nine circulating biomarkers reflecting nutritional, inflammatory, hematologic, renal, hepatic, and metabolic function (10, 11). Phenotypic Age Acceleration (PAA) is the deviation of Phenotypic Age from the value expected at a given chronological age. In an international analysis of more than 430,000 surgical patients, higher Phenotypic Age was associated with mortality, cardiovascular events, readmission, and early postoperative complications (12). Subsequent multicenter studies reported associations of accelerated biologic aging with postoperative acute kidney injury (13) and intraoperative hypotension (14). These findings raise the possibility that biologic aging captures aspects of perioperative reserve not represented by chronological age.

Laboratory Frailty Index (FI-Lab) approaches vulnerability differently by treating each abnormal laboratory result as a deficit and expressing the total as a proportion of measured deficits (15, 16). Higher FI-Lab values have been associated with mortality and other adverse outcomes in community cohorts across adulthood (17, 18). In surgical populations, preoperative FI-Lab has also been linked to mortality, intensive care use, longer hospitalization, readmission, and nonhome discharge (19, 20). In the present 17-item index, nine biomarkers are components of Phenotypic Age. The two measures therefore overlap biologically, although they weight abnormalities differently.

For perioperative use, association alone is insufficient. A marker also needs to retain its prognostic behavior across institutions and improve prediction beyond information that is already available clinically. Added value is not captured by discrimination alone: overall prediction error (21), calibration (22), and clinical net benefit (23) address different aspects of performance, while reclassification measures provide supplementary information about changes in risk separation (24). Small event counts can also inflate apparent performance and widen uncertainty, increasing the importance of shrinkage and independent validation (25). These issues are especially relevant to laboratory-derived markers because case mix, assay distributions, and baseline risk may vary across hospitals.

In this dual-center cohort, we assessed PAA and FI-Lab in harmonized data from the Second Affiliated Hospital of Shandong First Medical University and the Affiliated Taizhou People’s Hospital of Nanjing Medical University. The primary analyses examined their associations with all-cause mortality through 540 days and Shandong-to-Jiangsu external validation for 365-day mortality prediction; the reverse Jiangsu-to-Shandong direction was treated as a secondary reciprocal transportability sensitivity analysis because the PAA reference equation and standardization were derived from Shandong. Secondary analyses evaluated a 12-month composite of death or incident cardiometabolic-renal disease, and exploratory analyses described within-person changes in both indices. We expected the direction of mortality associations to be similar across centers; however, we did not assume that absolute fixed-reference PAA values would be directly interchangeable when center-specific age–Phenotypic Age relations differed.

## 2 Materials and methods

### 2.1 Study design and setting

We conducted a dual-center hospital-based cohort study using harmonized data collected during 2024–2025 from the health examination centers of the Second Affiliated Hospital of Shandong First Medical University and the Affiliated Taizhou People’s Hospital of Nanjing Medical University. Health-examination and structured-questionnaire information was linked to perioperative variables and subsequent follow-up. The laboratory biomarkers used to calculate PAA and FI-Lab were taken specifically from the preoperative laboratory panel obtained within 24 h before the index operation, rather than from an unspecified health-examination laboratory time point. The analytic cohort comprised 1,223 adults with complete baseline information required for the prespecified analyses: 671 participants from Shandong and 552 from Jiangsu. When more than one qualifying operation was recorded, the first qualifying procedure was treated as the index operation. Reporting followed the STROBE statement for observational studies (26). Prediction analyses were reported according to TRIPOD+AI principles (27).

Participants were eligible if they were aged 18 years or older, underwent a qualifying operation during the study period, completed the health-examination and structured-questionnaire data collection, had a complete laboratory panel required to calculate PAA and FI-Lab obtained within 24 h before the index operation, had complete prespecified perioperative covariates [American Society of Anesthesiologists (ASA) physical status, Charlson Comorbidity Index (CCI), FRAIL score, surgical stress level, and cancer-related surgery], and could be linked to postoperative mortality follow-up. Thus, availability of all required PAA/FI-Lab laboratory measurements within the 24-h preoperative window was an eligibility criterion. We excluded participants with no qualifying operation or an indeterminate index operation; age< 18 years or invalid age/surgery-date information; incomplete health-examination or questionnaire data; incomplete within-24-h preoperative laboratory data required for PAA or FI-Lab; incomplete perioperative covariates; or unavailable mortality-status/postoperative follow-up linkage. The initial candidate pools comprised 3,527 participants in Shandong and 3,411 in Jiangsu. After sequential screening, 671 and 552 participants, respectively, remained in the primary analytic cohort; the full selection process and the number excluded at each step are shown in **Figure 1**. Specifically, sequential exclusions in Shandong and Jiangsu were, respectively: 1,326 and 1,298 for no qualifying operation or an indeterminate index operation; 47 and 43 for age< 18 years or invalid age/surgery-date information; 582 and 606 for incomplete baseline health-examination/questionnaire data; 601 and 592 for incomplete PAA/FI-Lab laboratory variables; 176 and 192 for incomplete perioperative covariates; and 124 and 128 for unavailable mortality-status/postoperative follow-up linkage. The final analytic cohorts therefore represented 19.0% and 16.2% of the initial Shandong and Jiangsu candidate pools.

**Figure 1:**
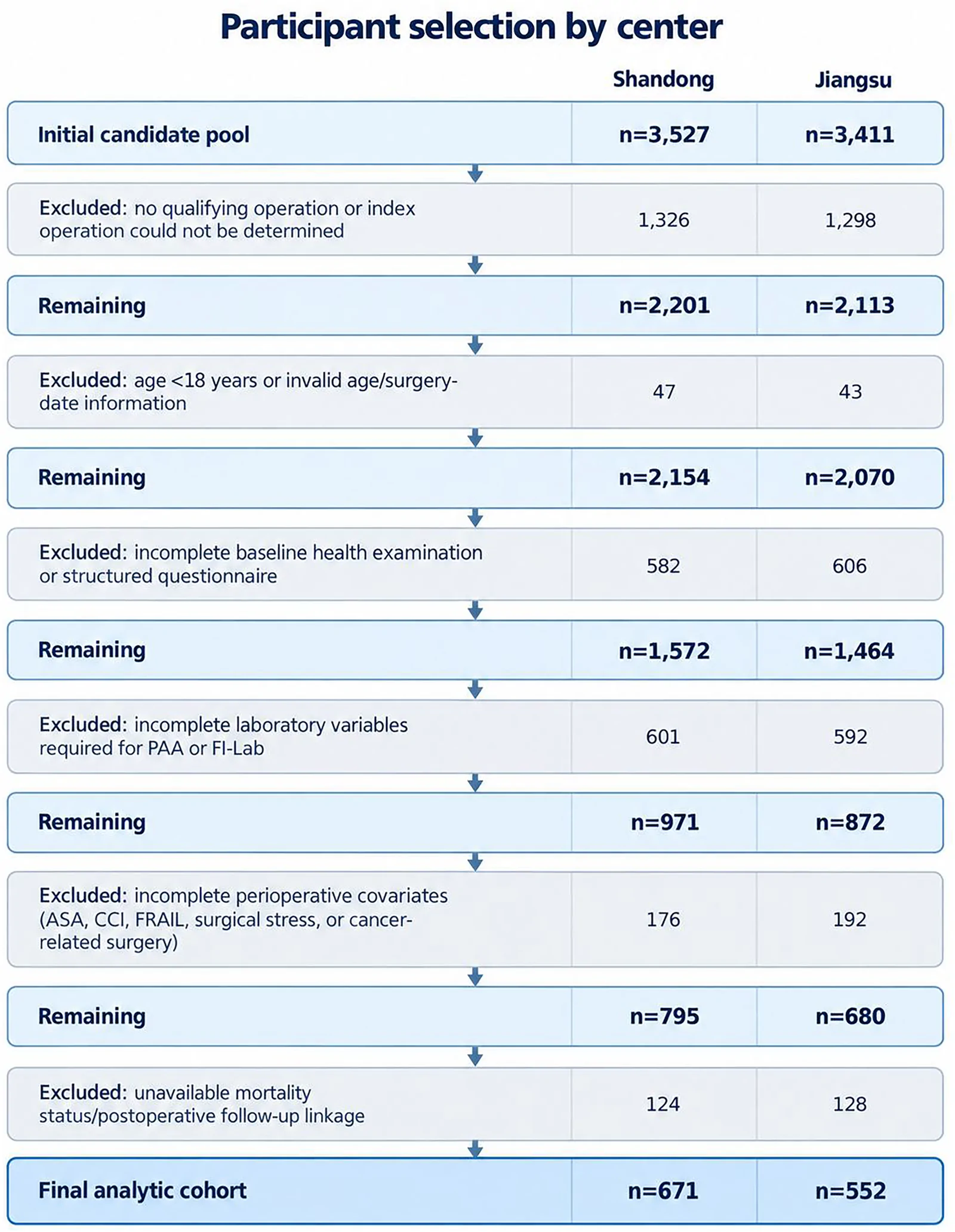
Participant-selection flow diagram for the Shandong and Jiangsu cohorts. Numbers show the sequential exclusions from the initial candidate pools to the final primary analytic cohorts.

### 2.2 Data collection and follow-up

Harmonized baseline variables included age, sex, body mass index, current smoking, hypertension, diabetes mellitus, chronic kidney disease (CKD), cardiovascular disease (CVD), ASA physical status, CCI, FRAIL score, surgical stress level, cancer-related surgery, and the laboratory measurements required for Phenotypic Age and FI-Lab. CCI was calculated without the age component using the original comorbidity framework (28). FRAIL was scored from its five questionnaire domains according to the published instrument (6). ASA physical status was retained as a prespecified clinical measure of perioperative disease burden (29, 30). Laboratory testing was performed in each hospital’s routine clinical laboratory under routine internal quality-control procedures. Before analysis, laboratory measurement units were harmonized across centers, and the centers’ routine reference intervals were broadly similar. Analyzer manufacturer/model and reagent-level metadata were not available in the present datasets; therefore, platform-specific recalibration could not be performed.

All laboratory measurements used for Phenotypic Age and FI-Lab were obtained within 24 h before the index operation. Surgical stress was categorized *a priori* as low/minor (Grade I), moderate (Grade II), or high/major (Grade III) according to expected invasiveness, physiologic disturbance, and blood loss. Low/minor procedures generally had expected blood loss< 500 mL and usually did not require postoperative ICU care; representative procedures included skin/subcutaneous procedures, breast biopsy, inguinal hernia repair, endoscopy/cystoscopy/hysteroscopy, limited laparoscopy, arthroscopy, and curettage. Moderate procedures were associated with more substantial tissue injury and inflammatory/metabolic stress and expected blood loss of 500–1,500 mL; representative procedures included cholecystectomy, thyroidectomy, nephrectomy, cystectomy, hysterectomy/myomectomy, laminectomy, hip or knee arthroplasty, and partial gastrointestinal resection/reconstruction. High/major procedures were highly invasive, with expected blood loss > 1,500 mL and frequent need for postoperative ICU care or invasive monitoring; representative procedures included intracranial, cardiothoracic, major vascular, extensive spine/orthopedic reconstruction, and major urologic reconstruction. Eligible operations spanned all three stress categories. These preoperative measurements were the laboratory values used for PAA and FI-Lab; complete availability within this 24-h window was required for cohort inclusion.

Vital status was followed from the index operation through 540 days. Fixed mortality horizons at 30, 90, 180, and 365 days were derived from the recorded follow-up interval and vital status. At approximately 12 months, disease status and laboratory measurements were updated when available. Incident diabetes, CKD, or CVD required the absence of that condition at baseline and new documentation at follow-up. CVD comprised coronary heart disease, myocardial infarction, stroke, or heart failure. The composite of death or incident diabetes, CKD, or CVD was prespecified to summarize clinically interrelated cardiovascular-kidney-metabolic outcomes (31, 32). Exact person-specific dates or intervals for the approximately 12-month laboratory reassessment were not available in the analytic datasets.

### 2.3 Phenotypic age and phenotypic age acceleration

Phenotypic Age was reconstructed identically in both centers from chronological age, serum albumin, creatinine, fasting glucose, C-reactive protein (CRP), lymphocyte percentage, mean corpuscular volume (MCV), red cell distribution width (RDW), alkaline phosphatase (ALP), and white blood cell count using the published mortality-score algorithm (10). CRP values recorded in milligrams per liter were divided by 10 before entry into the Phenotypic Age equation because the published algorithm specifies milligrams per deciliter; the original values in milligrams per liter were retained for FI-Lab classification. For the primary PAA definition, Shandong served as the reference population. Phenotypic Age was regressed on chronological age in Shandong, and the fitted equation was applied without re-estimation in Jiangsu. This kept the age-PhenoAge reference relation fixed across centers. Positive PAA indicates a Phenotypic Age older than expected from the Shandong age–PhenoAge relation. The resulting Shandong reference equation had an intercept of − 48.02 and an age coefficient of 1.66; PAA was standardized by the Shandong standard deviation (8.18 years) for regression and prediction analyses (12). We additionally estimated the baseline age–Phenotypic Age regression separately in each center and quantified the center-specific correlation between fixed-reference PAA and chronological age to assess dependence on the Shandong reference relation. As the age–Phenotypic Age relation could differ between centers, fixed-reference PAA was not assumed to be age-independent in Jiangsu; this dependence was examined explicitly through the center-specific age-PAA correlation and the center-specific residual sensitivity analysis.

The nine biomarkers were not selected *de novo* in the present cohorts. In the original Phenotypic Age development work, 42 routinely available clinical biomarkers together with chronological age were evaluated in NHANES III using a penalized Cox proportional-hazards model with 10-fold cross-validation; the final parsimonious mortality-prediction set comprised chronological age plus these nine biomarkers, and the resulting measure was subsequently validated in NHANES IV (10). We therefore applied the published biomarker panel and coefficients unchanged, rather than re-selecting biomarkers in our own data. To make the unit handling explicit, in both centers CRP values recorded in milligrams per liter were divided by 10 before entry into the Phenotypic Age equation, whereas the original values in milligrams per liter were retained for FI-Lab classification.


xb=−19.907−0.0336(albumin)+0.0095(creatinine)+0.1953(glucose)+0.0954 ln[CRP (mg/dL)]−0.0120(lymphocyte %)+0.0268(MCV)+0.3306(RDW)+0.00188(ALP)+0.0554(WBC)+0.0804(age)

(10)


Mortality score=1−exp{−[1.51714×exp(xb)]/0.0076927}

(10)


Phenotypic Age=141.50225+ln[−0.00553×ln(1−mortality score)]/0.090165

(10)

The primary acceleration measure was the residual from this fixed relation between Phenotypic Age and chronological age. Sensitivity analyses used center-specific residual definitions and center-specific standardization to assess whether results depended on the reference population (12). For the approximately 12-month reassessment, chronological age was set to baseline age +1.00 year before the fixed Shandong reference equation was applied to follow-up Phenotypic Age. A longitudinal sensitivity analysis instead used each center’s own baseline age–Phenotypic Age regression relation at both time points, again using baseline age + 1.00 year at follow-up. The center-specific residual analysis, with standardization performed within each center, was used as the key robustness analysis for determining whether the mortality association depended on the Shandong reference relation. As exact reassessment intervals were unavailable, the baseline age + 1.00-year convention for follow-up PAA should be regarded as an approximation for the exploratory longitudinal analysis.


$${\text{PAA}}_{i}={\text{Phenotypic Age}}_{i}-E({\text{Phenotypic Age}}_{i}|{\text{ chronological age}}_{i})$$

 (12)


$${\text{zPAA}}_{i}=\left({\text{PAA}}_{i}-\text{mean}\left[\text{PAA in Shandong}\right]\right)/\text{SD}\left(\text{PAA in Shandong}\right)$$

    (12)

### 2.4 Laboratory frailty index

FI-Lab was prespecified as the number of abnormal laboratory measurements divided by the total of 17 laboratory deficits, following the laboratory deficit-accumulation framework (15, 16). For primary analyses, the same fixed thresholds were used in both centers to avoid redefining frailty according to local distributions. FI-Lab was retained on its original 0-to-1 scale for descriptive and longitudinal analyses and standardized using the Shandong mean (0.11) and standard deviation (0.10) for regression and transportability analyses.

Under this prespecified deficit-accumulation formulation, an out-of-range result contributed one deficit regardless of whether the value was below or above the reference interval. This binary coding is intentional: FI-Lab is designed to quantify the burden of physiologic abnormalities rather than to assign direction-specific mortality weights. No center-specific or data-derived weights for low versus high abnormalities were estimated in the present study.


$${\text{FI-Lab}}_{i}=\Sigma_{j}I\left(X_{ij}\text{ outside the prespecified reference interval}\right)/17$$

(15, 16)

The 17 deficits were albumin< 40 or > 55 g/L; creatinine< 57 or > 111 μmol/L in men and< 41 or > 81 μmol/L in women; fasting glucose< 3.9 or > 6.1 mmol/L; CRP > 5 mg/L; lymphocyte percentage< 20% or > 50%; MCV< 82 or > 100 fL; RDW< 11.5% or > 14.5%; ALP< 45 or > 125 U/L; white blood cell count< 3.5 or > 9.5 ×10^9^/L; hemoglobin< 130 or > 175 g/L in men and< 115 or > 150 g/L in women; platelet count< 125 or > 350 ×10^9^/L; alanine aminotransferase< 7 or > 40 U/L; aspartate aminotransferase< 13 or > 35 U/L; total bilirubin< 5 or > 21 μmol/L; urea< 3.1 or > 8.0 mmol/L; sodium< 137 or > 147 mmol/L; and potassium< 3.5 or > 5.3 mmol/L. Each measurement contributed 0 when within and 1 when outside its prespecified interval.

### 2.5 Outcomes

The primary association outcome was all-cause mortality from the index operation through 540 days. The primary prediction endpoint was 365-day all-cause mortality. Mortality at 30, 90, and 180 days was examined as secondary fixed-horizon outcomes, consistent with the use of standardized perioperative mortality horizons (33).

The annual composite outcome was death within 365 days or incident diabetes, CKD, or CVD at the approximately 12-month update among participants free of all three diseases at baseline and with known outcome status. Each incident disease was also evaluated separately among participants without that condition at baseline. Exploratory longitudinal outcomes were within-person changes in PAA and FI-Lab between baseline and the approximately 12-month reassessment.

### 2.6 Statistical analysis and cross-center validation

Continuous variables are reported as mean [standard deviation (SD)], and categorical variables as number (percentage). Baseline characteristics were compared between centers using Welch *t*-tests for continuous variables and Chi-square or Fisher exact tests for categorical variables. Pearson correlation quantified the relation between PAA and FI-Lab. Effect estimates and descriptive statistics are reported to two decimal places; *p*-values are reported to two decimal places or as *p<* 0.01. All tests were two-sided. Baseline variables required for the primary analyses were complete in both cohorts, so multiple imputation was not required.

Center-specific Cox proportional hazards models estimated associations with mortality through 540 days, with PAA and FI-Lab modeled per 1-SD increase. Model 1 adjusted for age and sex. Model 2 additionally included ASA physical status, CCI, surgical stress level, and cancer-related surgery. Model 3 further included the FRAIL score. Pooled analyses used a center-stratified Cox model to permit center-specific baseline hazards. Center-by-exposure product terms were used to test cross-center heterogeneity, and a joint Model 3 included PAA and FI-Lab simultaneously. Restricted cubic splines evaluated dose–response shape, with three spline degrees of freedom and age-, sex-, and center-stratified adjustment for graphical analyses (34, 35).

For descriptive survival analyses, PAA was dichotomized at 0 years and FI-Lab at three abnormal measurements, generating four joint categories. Kaplan–Meier curves were compared with a global log-rank test. Fixed-horizon mortality analyses used logistic regression adjusted for age, sex, and center and were considered secondary because early-event counts were limited.

Incremental prediction was evaluated for 365-day mortality. The study clinical model included age, sex, ASA physical status, CCI, FRAIL score, surgical stress, and cancer-related surgery; extended models added PAA, FI-Lab, or both. Ridge logistic regression was tuned by threefold inner cross-validation. Internal performance was estimated with nested fivefold cross-validation. For the primary transportability analysis, the model was trained and tuned only in Shandong and then applied to Jiangsu without refitting; reciprocal Jiangsu-to-Shandong validation was prespecified as a secondary analysis. Keeping model development and evaluation in separate centers reduced reuse of the validation data (25, 27).

As the PAA reference equation and the exposure standardization parameters were derived from Shandong, the Jiangsu-to-Shandong direction was prespecified and interpreted as a secondary reciprocal transportability sensitivity analysis, not as a fully independent external validation. The primary external-validation direction remained Shandong-to-Jiangsu.

Discrimination and overall prediction error were evaluated using the AUC, precision-recall AUC (PR-AUC), and Brier score (21). Calibration was summarized by the calibration intercept, calibration slope, and calibration plots (22). Interpretation of discrimination and calibration followed established guidance for clinical prediction models (36). Continuous net reclassification improvement (NRI) and integrated discrimination improvement (IDI) were secondary measures of incremental separation (24). Decision-curve analysis quantified net benefit across clinically plausible threshold probabilities (23). Confidence intervals and *p*-values for paired differences between models were obtained from 1,000 stratified bootstrap samples. Wald 95% confidence intervals were also reported for the calibration intercept and calibration slope in the primary external-validation cohort. For overall interpretation of incremental prediction, discrimination (AUC and PR-AUC), overall prediction error (Brier score), calibration, and decision-curve net benefit were prioritized. NRI and IDI were treated as supplementary reclassification measures and were not considered evidence of clinical utility in isolation.

Given the limited number of validation events, calibration-intercept and calibration-slope hypothesis tests were treated as descriptive. In particular, failure to reject an intercept of 0 or a slope of 1 was not interpreted as evidence of adequate calibration. Given the sparse validation events, effect estimates and 95% confidence intervals were emphasized over dichotomous interpretation of nonsignificant *p*-values.

Modified Poisson regression with robust variance estimated risk ratios for the annual composite and individual incident diseases (37). The fully adjusted composite model included age, sex, body mass index, smoking, hypertension, ASA physical status, CCI, FRAIL score, surgical stress, cancer-related surgery, and center. Disease-specific models were adjusted for age, sex, and center, with false-discovery-rate correction across the six exposure-outcome tests. Paired *t*-tests assessed annual within-person changes. Stabilized inverse-probability weights were used as a sensitivity analysis for selective availability of repeat laboratory assessment, with the observation model including baseline demographics, comorbidities, surgical variables, center, PAA, and FI-Lab (38).

Sensitivity analyses repeated the fully adjusted center-stratified mortality models using center-specific PAA residual definitions together with center-specific exposure standardization, and after excluding deaths within 30 days. Analyses were conducted in Python 3.13 using NumPy, SciPy, statsmodels, lifelines, and scikit-learn. For longitudinal PAA, the center-specific-residual sensitivity analysis applied each center’s own baseline age–Phenotypic Age equation at baseline and follow-up.

## 3 Results

### 3.1 Cohort characteristics and follow-up

The study included 1,223 participants: 671 from Shandong and 552 from Jiangsu. The mean age was 59.37 (10.92) years, and 643 participants (52.58%) were men. The two centers were similar in age [59.38 (11.37) vs. 59.35 (10.35) years; *p* = 0.96], CCI [1.18 (1.41) vs. 1.09 (1.20); *p* = 0.22], FRAIL score [0.71 (1.29) vs. 0.64 (0.80); *p* = 0.30], and PAA [0.00 (8.18) vs. − 0.58 (9.54) years; *p* = 0.26]. FI-Lab was higher in Shandong than in Jiangsu [0.11 (0.10) vs. 0.06 (0.06); *p<* 0.01], while most other baseline characteristics showed no material center difference (**Table 1**).

**Table 1:** Baseline characteristics by center.

Characteristic	Overall	Shandong	Jiangsu	*P*-value
Overall	1,223	671	552	
Age (years)	59.37 (10.92)	59.38 (11.37)	59.35 (10.35)	0.96
Body mass index (kg/m^2^)	24.32 (2.36)	24.35 (2.58)	24.28 (2.04)	0.56
Charlson Comorbidity Index	1.14 (1.32)	1.18 (1.41)	1.09 (1.20)	0.22
FRAIL score	0.68 (1.10)	0.71 (1.29)	0.64 (0.80)	0.30
Albumin (g/L)	43.95 (3.11)	43.95 (3.33)	43.95 (2.83)	1.00
Creatinine (μmol/L)	70.19 (16.31)	70.27 (18.48)	70.09 (13.22)	0.85
Fasting glucose (mmol/L)	5.31 (1.08)	5.31 (1.21)	5.30 (0.89)	0.91
C-reactive protein (mg/L)	3.01 (3.84)	3.11 (4.19)	2.89 (3.38)	0.30
Lymphocyte percentage (%)	32.74 (6.56)	32.69 (7.35)	32.80 (5.45)	0.77
Mean corpuscular volume (fL)	90.61 (3.92)	90.55 (4.12)	90.70 (3.66)	0.49
Red cell distribution width (%)	12.77 (1.26)	12.86 (1.48)	12.66 (0.91)	< 0.01
Alkaline phosphatase (U/L)	80.51 (24.22)	79.75 (26.12)	81.42 (21.67)	0.22
White blood cell count (× 10^9^/L)	6.10 (1.00)	6.08 (1.04)	6.12 (0.94)	0.42
Hemoglobin (g/L)	138.88 (13.06)	139.50 (14.08)	138.13 (11.69)	0.06
Platelet count (× 10^9^/L)	220.27 (37.66)	221.70 (39.25)	218.52 (35.59)	0.14
Alanine aminotransferase (U/L)	23.05 (9.52)	22.83 (9.39)	23.32 (9.68)	0.37
Aspartate aminotransferase (U/L)	22.86 (6.79)	22.92 (7.55)	22.79 (5.74)	0.73
Total bilirubin (μmol/L)	12.88 (3.71)	12.77 (3.88)	13.01 (3.47)	0.25
Urea (mmol/L)	4.97 (1.36)	5.01 (1.46)	4.92 (1.22)	0.20
Sodium (mmol/L)	141.03 (1.27)	141.05 (1.33)	141.01 (1.19)	0.54
Potassium (mmol/L)	4.17 (0.31)	4.16 (0.32)	4.17 (0.29)	0.62
Phenotypic Age (years)	50.14 (17.21)	50.42 (20.55)	49.80 (11.97)	0.51
Phenotypic Age Acceleration (years)	− 0.26 (8.82)	0.00 (8.18)	− 0.58 (9.54)	0.26
Laboratory Frailty Index	0.09 (0.09)	0.11 (0.10)	0.06 (0.06)	< 0.01
Sex				
Female	580 (47.42)	311 (46.35)	269 (48.73)	0.44
Male	643 (52.58)	360 (53.65)	283 (51.27)	
Current smoking				
No	992 (81.11)	547 (81.52)	445 (80.62)	0.74
Yes	231 (18.89)	124 (18.48)	107 (19.38)	
Hypertension				
No	755 (61.73)	407 (60.66)	348 (63.04)	0.43
Yes	468 (38.27)	264 (39.34)	204 (36.96)	
Diabetes mellitus				
No	1,055 (86.26)	569 (84.80)	486 (88.04)	0.12
Yes	168 (13.74)	102 (15.20)	66 (11.96)	
Chronic kidney disease				
No	1,109 (90.68)	598 (89.12)	511 (92.57)	0.05
Yes	114 (9.32)	73 (10.88)	41 (7.43)	
Cardiovascular disease				
No	1,088 (88.96)	594 (88.52)	494 (89.49)	0.66
Yes	135 (11.04)	77 (11.48)	58 (10.51)	
ASA physical status				
I	159 (13.00)	81 (12.07)	78 (14.13)	0.59
II	676 (55.27)	368 (54.84)	308 (55.80)	
III	341 (27.88)	195 (29.06)	146 (26.45)	
IV	47 (3.84)	27 (4.02)	20 (3.62)	
Surgical stress level				
Low	237 (19.38)	126 (18.78)	111 (20.11)	0.50
Moderate	672 (54.95)	364 (54.25)	308 (55.80)	
High	314 (25.67)	181 (26.97)	133 (24.09)	
Cancer-related surgery				
No	916 (74.90)	489 (72.88)	427 (77.36)	0.08
Yes	307 (25.10)	182 (27.12)	125 (22.64)	

Values are means (SD) or *n* (%). *p*-values for comparisons between Shandong and Jiangsu were obtained using Welch *t*-tests for continuous variables and Chi-square or Fisher exact tests for categorical variables. PAA was defined using the fixed Shandong age—Phenotypic Age reference; because this definition was strongly age-dependent in Jiangsu, the unadjusted between-center PAA comparison is descriptive and should not be interpreted as an age-independent center effect.

As shown in **Figure 1**, screening began with 3,527 candidate participants in Shandong and 3,411 in Jiangsu. In Shandong, 1,326 were excluded for no qualifying operation or an indeterminate index operation, 47 for age/date criteria, 582 for incomplete baseline examination/questionnaire data, 601 for incomplete PAA/FI-Lab laboratory data, 176 for incomplete perioperative covariates, and 124 for unavailable mortality follow-up, leaving 671 participants (19.0% of the initial candidate pool). In Jiangsu, the corresponding exclusions were 1,298, 43, 606, 592, 192, and 128, leaving 552 participants (16.2% of the initial candidate pool).

Over 1,694.66 person-years, 74 participants died (39 in Shandong and 35 in Jiangsu), corresponding to 4.37 deaths per 100 person-years. Deaths numbered 14 by 30 days, 24 by 90 days, 36 by 180 days, 57 by 365 days, and 74 by 540 days; the corresponding pooled Kaplan–Meier survival estimates were 98.86%, 98.04%, 97.06%, 95.34%, and 93.86%. Overall survival did not differ between centers (log-rank *p* = 0.81).

### 3.2 Cross-center associations with mortality

In Shandong, PAA was associated with mortality in Models 1 and 2 [hazard ratio (HR), 1.54; 95% confidence interval (CI), 1.14–2.08; *p<* 0.01 and HR, 1.42; 95% CI, 1.03–1.96; *p* = 0.03] but attenuated after FRAIL was added (HR, 1.29; 95% CI, 0.91–1.82; *p* = 0.15). In Jiangsu, the association remained after full adjustment (HR, 1.77; 95% CI, 1.11–2.84; *p* = 0.02). FI-Lab showed a similar center contrast: the fully adjusted HR was 1.16 (95% CI, 0.86–1.56; *p* = 0.34) in Shandong and 1.89 (95% CI, 1.19–3.02; *p<* 0.01) in Jiangsu (**Table 2**).

**Table 2:** Center-specific and pooled associations of PAA and FI-Lab with all-cause mortality through 540 days.

Exposure	Center/model	*N*	Events	HR (95% CI)	*P*-value
PAA	Shandong/Model 1	671	39	1.54 (1.14–2.08)	< 0.01
PAA	Shandong/Model 2	671	39	1.42 (1.03–1.96)	0.03
PAA	Shandong/Model 3	671	39	1.29 (0.91–1.82)	0.15
FI-Lab	Shandong/Model 1	671	39	1.39 (1.07–1.80)	0.01
FI-Lab	Shandong/Model 2	671	39	1.31 (0.99–1.72)	0.05
FI-Lab	Shandong/Model 3	671	39	1.16 (0.86–1.56)	0.34
PAA	Jiangsu/Model 1	552	35	2.13 (1.47–3.09)	< 0.01
PAA	Jiangsu/Model 2	552	35	1.81 (1.20–2.74)	< 0.01
PAA	Jiangsu/Model 3	552	35	1.77 (1.11–2.84)	0.02
FI-Lab	Jiangsu/Model 1	552	35	2.30 (1.56–3.40)	< 0.01
FI-Lab	Jiangsu/Model 2	552	35	1.98 (1.29–3.05)	< 0.01
FI-Lab	Jiangsu/Model 3	552	35	1.89 (1.19–3.02)	< 0.01
PAA	Pooled/Model 1	1,223	74	1.69 (1.36–2.09)	< 0.01
PAA	Pooled/Model 2	1,223	74	1.48 (1.17–1.87)	< 0.01
PAA	Pooled/Model 3	1,223	74	1.34 (1.03–1.75)	0.03
FI-Lab	Pooled/Model 1	1,223	74	1.61 (1.31–1.99)	< 0.01
FI-Lab	Pooled/Model 2	1,223	74	1.43 (1.14–1.78)	< 0.01
FI-Lab	Pooled/Model 3	1,223	74	1.28 (1.00–1.65)	0.05

Hazard ratios are per 1-SD increase in exposure. Model 1 was adjusted for age and sex. Model 2 was additionally adjusted for ASA physical status, Charlson Comorbidity Index, surgical stress level, and cancer-related surgery. Model 3 was additionally adjusted for FRAIL score. Pooled models were stratified by center.

In pooled center-stratified analyses, PAA remained associated with mortality after full adjustment (HR, 1.34 per SD; 95% CI, 1.03–1.75; *p* = 0.03). The FI-Lab estimate was in the same direction but borderline (HR, 1.28 per SD; 95% CI, 1.00–1.65; *p* = 0.05). There was no evidence that the PAA association differed by center (interaction ratio, 1.06; 95% CI, 0.68–1.65; *p* = 0.79), whereas the FI-Lab interaction suggested possible but nonsignificant heterogeneity (interaction ratio, 1.48; 95% CI, 0.94–2.32; *p* = 0.09). When both indices were entered simultaneously, neither retained a statistically significant independent association (PAA HR, 1.25; 95% CI, 0.94–1.65; *p* = 0.12; FI-Lab HR, 1.19; 95% CI, 0.90–.57; *p* = 0.22) (**Table 3**). Given that the fixed-reference PAA retained substantial age dependence in Jiangsu, we additionally emphasize the center-specific-residual sensitivity analysis: after recalculating PAA from each center’s own age–Phenotypic Age relation and standardizing exposures within each center, the fully adjusted pooled PAA association had an HR of 1.39 (95% CI, 1.09–1.77), supporting the robustness of the association while not implying interchangeability of absolute fixed-reference PAA values.

**Table 3:** Joint associations and cross-center heterogeneity of PAA and FI-Lab with all-cause mortality through 540 days.

Measure	Analysis	*N*	Events	Effect (95% CI)	*P*-value
PAA	Joint Model 3	1,223	74	1.25 (0.94–1.65)	0.12
FI-Lab	Joint Model 3	1,223	74	1.19 (0.90–1.57)	0.22
PAA × center	Fully adjusted interaction	1,223	74	1.06 (0.68–1.65)	0.79
FI-Lab × center	Fully adjusted interaction	1,223	74	1.48 (0.94–2.32)	0.09
PAA vs. FI-Lab	Pearson correlation	1,223	–	*r* = 0.25	< 0.01

The joint model contained PAA and FI-Lab simultaneously and was adjusted for all Model 3 covariates. Interaction ratios are multiplicative center-by-exposure terms; Shandong was the reference center. Pearson correlation was calculated in the pooled cohort.

Age- and sex-adjusted restricted cubic splines were compatible with approximately linear risk gradients for both indices (PAA: overall *p<* 0.01, nonlinear *p* = 0.62; FI-Lab: overall *p<* 0.01, nonlinear *p* = 0.68) (**Figure 2**). Survival differed across the four joint PAA/FI-Lab categories (global log-rank *p<* 0.01), with the lowest survival among participants with PAA > 0 and at least three laboratory deficits (**Figure 3**).

**Figure 2:**
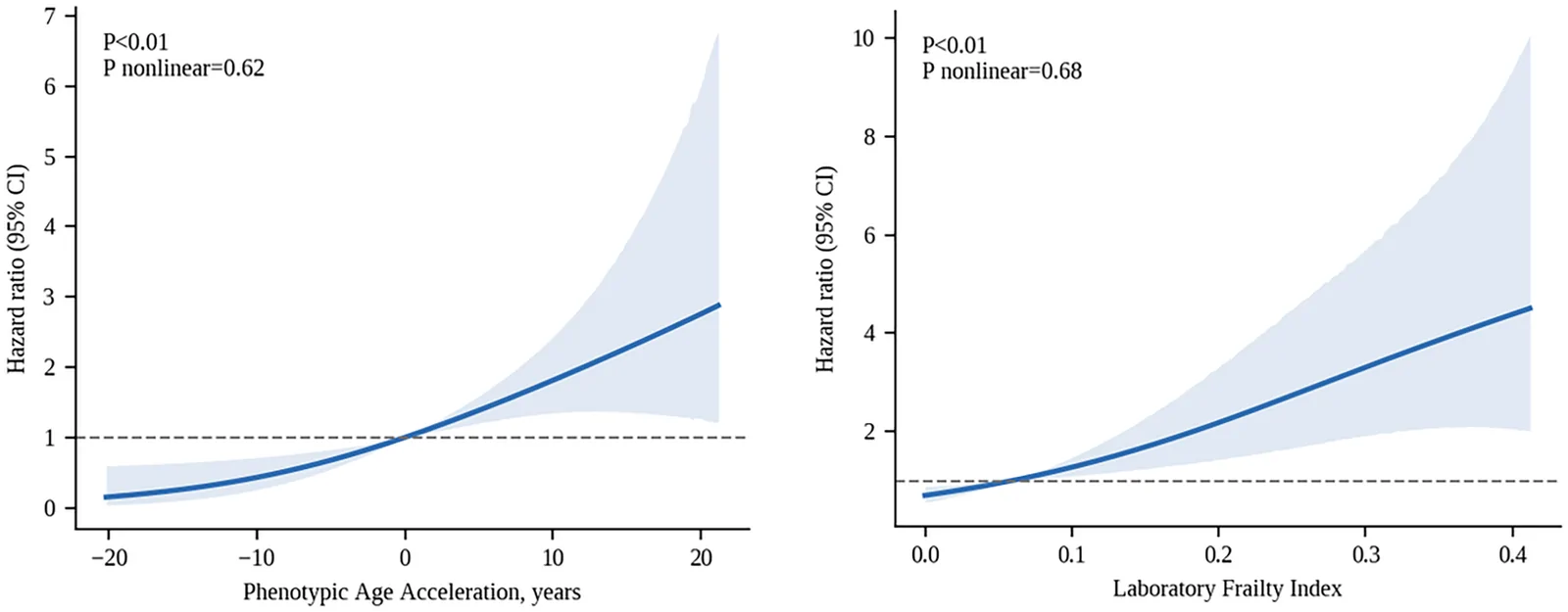
Restricted cubic spline associations of PAA and FI-Lab with all-cause mortality through 540 days. Curves are from age- and sex-adjusted center-stratified Cox models. For PAA, overall *p<* 0.01 and *p* for nonlinearity = 0.62. For FI-Lab, overall *p<* 0.01 and *p* for nonlinearity = 0.68. Shaded regions indicate 95% CIs.

**Figure 3:**
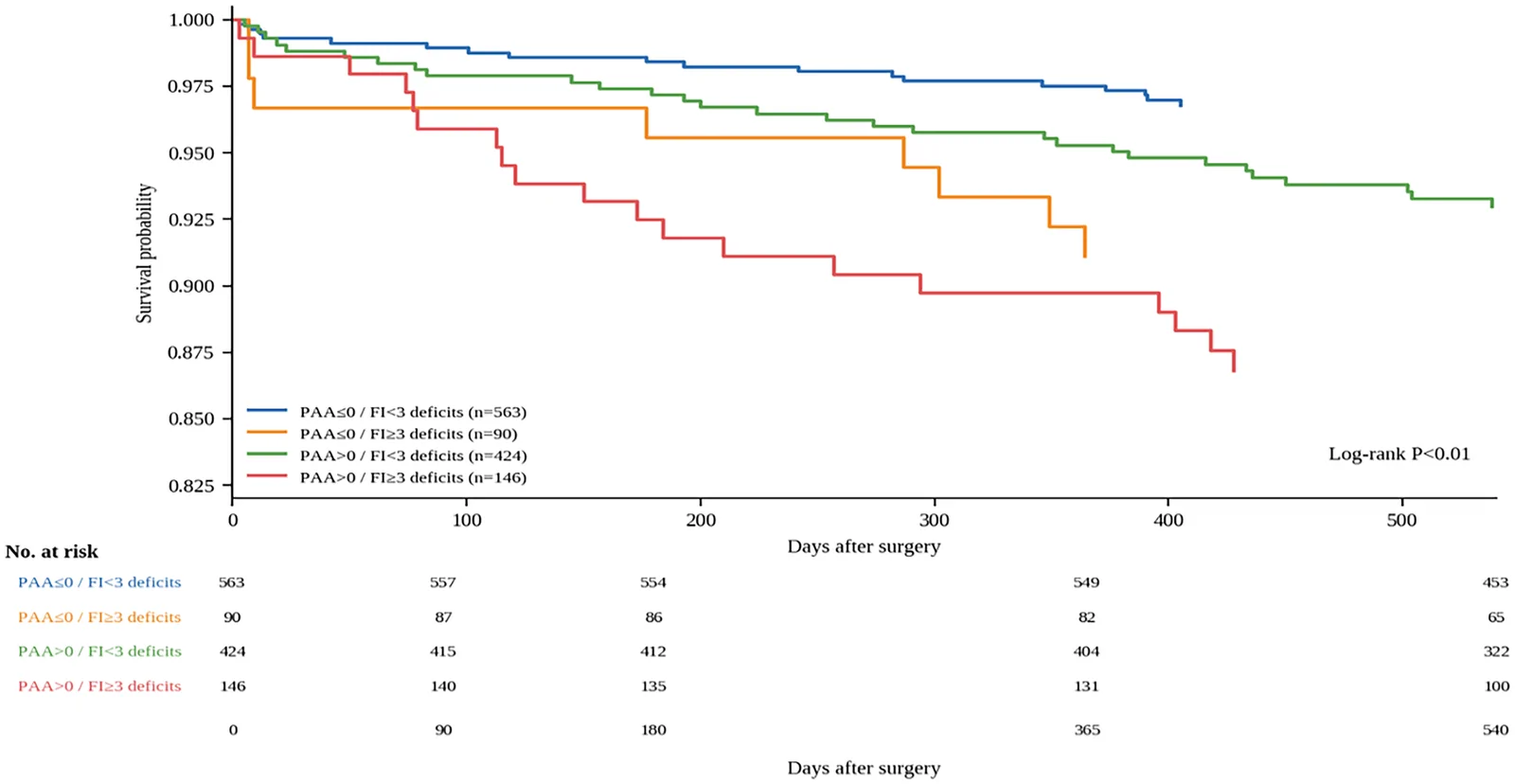
Kaplan–Meier survival curves according to joint PAA and FI-Lab categories in the pooled cohort. PAA was classified at 0 years and FI-Lab at three abnormal laboratory measurements. The global log-rank *p<* 0.01. Numbers at risk are shown beneath the curves. As fixed-reference PAA was age-dependent in Jiangsu, these unadjusted curves are descriptive; age-adjusted Cox models are the primary inferential analyses.

### 3.3 External validation, secondary reciprocal transportability analysis, and incremental prediction

The Shandong-to-Jiangsu analysis was the primary external validation. The Jiangsu-to-Shandong analysis was considered a secondary reciprocal transportability sensitivity analysis because the PAA reference equation and standardization were derived from Shandong.

In the primary Shandong-to-Jiangsu external validation, the locked study clinical model had an AUC of 0.60 (95% CI, 0.45–0.73), a PR-AUC of 0.12 (95% CI, 0.05–0.26), and a Brier score of 0.04. Adding PAA produced an AUC of 0.60 (95% CI, 0.48–0.73; AUC difference, 0.00; 95% CI, − 0.04 to 0.05). Adding FI-Lab produced an AUC of 0.63 (95% CI, 0.49–0.76; difference, 0.03; 95% CI, − 0.01 to 0.09), and adding both yielded an AUC of 0.63 (95% CI, 0.49–0.75; difference, 0.03; 95% CI, − 0.02 to 0.10) (**Tables 4**, **5**, **Figure 4**). Calibration estimates were imprecise because only 23 deaths occurred by 365 days in Jiangsu. Calibration intercepts (95% CIs) were − 0.18 (− 0.59 to 0.23) for the study clinical model, − 0.19 (− 0.61 to 0.23) for the study clinical model + PAA, − 0.04 (− 0.43 to 0.35) for the study clinical model + FI-Lab, and − 0.12 (− 0.55 to 0.31) for the combined model. Corresponding calibration slopes were 2.61 (0.09 to 5.13), 4.38 (0.47 to 8.29), 1.22 (0.30 to 2.14), and 2.01 (0.50 to 3.52). The wide intervals show substantial uncertainty; all slope intervals included one. Accordingly, nonsignificant calibration tests were not interpreted as evidence of adequate calibration.

**Table 4:** Primary external validation and secondary reciprocal transportability performance for 365-day mortality.

Validation direction	Model	AUC (95% CI)	*p*-value	PR-AUC (95% CI)	*p*-value	Brier score (*p*-value)	Calibration intercept (*p*-value)	Calibration slope (*p*-value)
Shandong → Jiangsu	Study clinical model	0.60 (0.45–0.73)	0.16	0.12 (0.05–0.26)	<⁠ 0.⁠01	0.04 (0.35)	− 0.18 (0.39)	2.61 (0.21)
Shandong → Jiangsu	Study clinical model + PAA	0.60 (0.48–0.73)	0.12	0.10 (0.05–0.26)	0.01	0.04 (0.37)	− 0.19 (0.37)	4.38 (0.09)
Shandong → Jiangsu	Study clinical model + FI-Lab	0.63 (0.49–0.76)	0.07	0.11 (0.06–0.24)	< ⁠0.⁠01	0.04 (0.18)	− 0.04 (0.84)	1.22 (0.64)
Shandong → Jiangsu	Study clinical model + PAA + FI-Lab	0.63 (0.49–0.75)	0.06	0.11 (0.05–0.25)	< ⁠0.⁠01	0.04 (0.14)	− 0.12 (0.58)	2.01 (0.19)
Jiangsu → Shandong	Study clinical model	0.66 (0.57–0.75)	< 0.01	0.11 (0.07–0.19)	<⁠0.⁠01	0.05 (0.85)	0.12 (0.52)	0.64 (0.06)
Jiangsu → Shandong	Study clinical model + PAA	0.69 (0.58–0.79)	< ⁠0.⁠01	0.13 (0.09–0.21)	<⁠ 0.⁠01	0.05 (0.31)	− 0.25 (0.18)	0.49 (<⁠ 0.⁠01)
Jiangsu → Shandong	Study clinical model + FI-Lab	0.67 (0.57–0.77)	<⁠ 0.⁠01	0.12 (0.08–0.21)	< ⁠0.⁠01	0.05 (0.19)	− 0.38 (0.05)	0.54 (< ⁠0.⁠01)
Jiangsu → Shandong	Study clinical model + PAA + FI-Lab	0.67 (0.57–0.77)	< ⁠0.⁠01	0.12 (0.08–0.21)	<⁠ 0.⁠01	0.06 (0.04)	− 0.57 (< ⁠0.⁠01)	0.47 (< ⁠0.⁠01)

The study clinical model included age, sex, ASA physical status, Charlson Comorbidity Index, FRAIL score, surgical stress, and cancer-related surgery. *p*-values for the AUC compare with 0.50; *p*-values for the PR-AUC compare with outcome prevalence; Brier *p*-values compare the Brier score with a constant-risk no-information model; calibration *p*-values compare the intercept with 0 and the slope with 1. Shandong-to-Jiangsu was the primary external validation; Jiangsu-to-Shandong was a secondary transportability sensitivity analysis because the PAA reference equation and standardization were derived from Shandong. Calibration *p*-values are descriptive; because only 23 deaths occurred in the primary validation cohort, calibration should be interpreted from point estimates and 95% CIs.

**Table 5:** Incremental value of PAA and FI-Lab relative to the study clinical model in the primary Shandong-to-Jiangsu external validation.

Extended model	Measure	Estimate (95% CI)	*p*-value
Study clinical model + PAA	AUC difference	0.00 (− 0.04, 0.05)	0.79
Study clinical model + PAA	PR-AUC difference	− 0.02 (− 0.05, 0.02)	0.80
Study clinical model + PAA	Brier difference	0.00 (0.00, 0.00)	0.35
Study clinical model + PAA	Continuous NRI	− 0.15 (− 0.57, 0.26)	0.48
Study clinical model + PAA	IDI	0.00 (0.00, 0.00)	0.18
Study clinical model + FI-Lab	AUC difference	0.03 (− 0.01, 0.09)	0.10
Study clinical model + FI-Lab	PR-AUC difference	− 0.02 (− 0.05, 0.03)	0.89
Study clinical model + FI-Lab	Brier difference	0.00 (0.00, 0.00)	0.17
Study clinical model + FI-Lab	Continuous NRI	0.57 (0.19, 0.99)	< 0.01
Study clinical model + FI-Lab	IDI	0.01 (0.00, 0.02)	0.02
Study clinical model + PAA + FI-Lab	AUC difference	0.03 (− 0.02, 0.10)	0.31
Study clinical model + PAA + FI-Lab	PR-AUC difference	− 0.01 (− 0.05, 0.04)	0.94
Study clinical model + PAA + FI-Lab	Brier difference	0.00 (0.00, 0.00)	0.07
Study clinical model + PAA + FI-Lab	Continuous NRI	0.45 (0.05, 0.87)	0.03
Study clinical model + PAA + FI-Lab	IDI	0.00 (0.00, 0.01)	0.03

Estimates, 95% CIs, and *p*-values were obtained from 1,000 paired stratified bootstrap samples. Positive AUC, PR-AUC, NRI, and IDI differences and negative Brier score differences favor the extended model. NRI and IDI are supplementary reclassification metrics; overall interpretation of incremental performance prioritizes AUC/PR-AUC, Brier score, calibration, and decision-curve net benefit.

**Figure 4:**
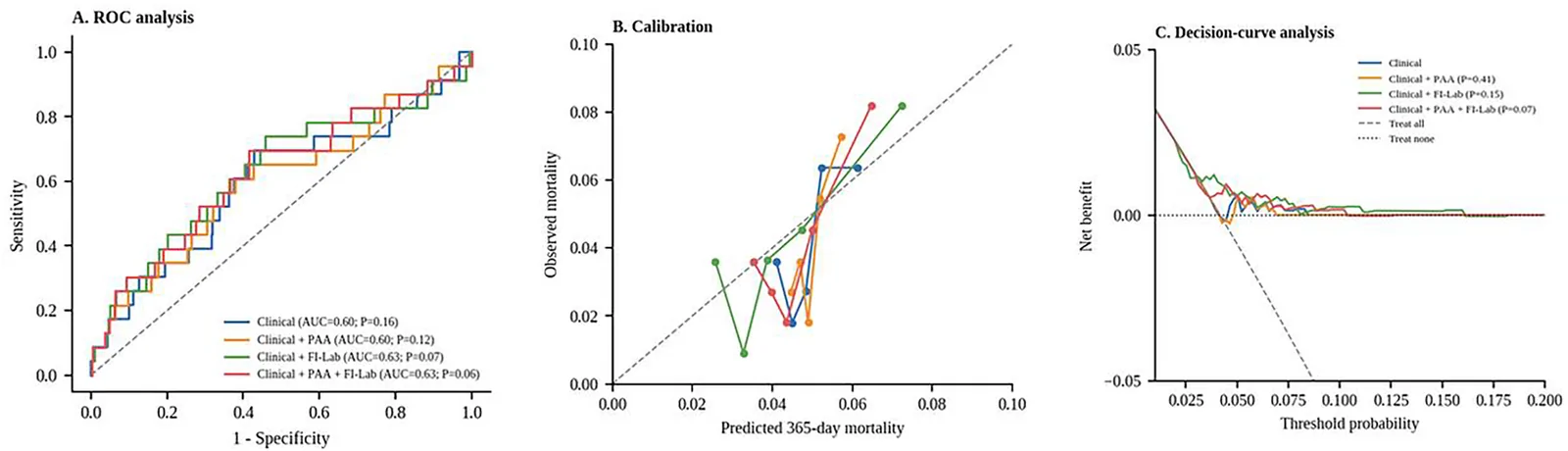
Primary Shandong-to-Jiangsu external validation for 365-day mortality. **(A)** Receiver operating characteristic curves with AUC and *p*-values versus 0.50. **(B)** Calibration by risk quintile; the dashed line indicates perfect calibration. **(C)** Decision-curve analysis; *p*-values in the legend compare mean net benefit with that of the study clinical model over prespecified clinically relevant thresholds.

In the primary external validation, supplementary reclassification metrics favored FI-Lab (continuous NRI, 0.57; 95% CI, 0.19–0.99; IDI, 0.01; 95% CI, 0.00–0.02) and the combined model (NRI, 0.45; 95% CI, 0.05–0.87; IDI, 0.00; 95% CI, 0.00–0.01), but these findings were not accompanied by consistent improvement in AUC, PR-AUC, Brier score, calibration, or decision-curve net benefit (**Table 5**; **Figure 4**). We therefore did not interpret NRI or IDI in isolation as evidence of improved transportable prediction. Decision-curve analysis showed no consistent net-benefit improvement over the study clinical model across the prespecified thresholds. In the secondary reciprocal Jiangsu-to-Shandong transportability analysis, the study clinical model AUC was 0.66, and the AUCs after adding PAA, FI-Lab, or both were 0.69, 0.67, and 0.67; however, calibration slopes for the extended models were below 1, again indicating center-dependent calibration (**Table 4**).

### 3.4 Annual cardiometabolic-renal outcomes

Among 815 participants free of diabetes, CKD, and CVD at baseline with known annual outcome status, 101 died or developed at least 1 of these conditions by 12 months. FI-Lab was associated with the composite after full adjustment [relative risk (RR), 1.36 per SD; 95% CI, 1.08–1.71; *p* = 0.01], whereas PAA was not (RR, 1.17; 95% CI, 0.92–1.48; *p* = 0.19). In the joint model, FI-Lab remained associated with risk (RR, 1.33; 95% CI, 1.05–1.69; *p* = 0.02), while PAA did not (RR, 1.11; 95% CI, 0.90–1.37; *p* = 0.33) (**Table 6**).

**Table 6:** Associations of PAA and FI-Lab with 12-month death or incident cardiometabolic-renal disease.

Exposure	Model	*N*	Events	RR (95% CI)	*p*-value
PAA	Fully adjusted	815	101	1.17 (0.92–1.48)	0.19
FI-Lab	Fully adjusted	815	101	1.36 (1.08–1.71)	< 0.01
PAA	Joint fully adjusted	815	101	1.11 (0.90–1.37)	0.33
FI-Lab	Joint fully adjusted	815	101	1.33 (1.05–1.69)	0.02

Risk ratios are per 1-SD higher exposure. Fully adjusted models included age, sex, body mass index, smoking, hypertension, ASA physical status, Charlson Comorbidity Index, FRAIL score, surgical stress, cancer-related surgery, and center. Joint models contained both PAA and FI-Lab.

Disease-specific analyses included 28 incident diabetes events, 34 incident CKD events, and 29 incident CVD events. PAA and FI-Lab were each associated with incident diabetes after false-discovery-rate (FDR) correction (PAA RR, 1.77; 95% CI, 1.31–2.40; FDR *p<* 0.01; FI-Lab RR, 1.66; 95% CI, 1.24–2.23; FDR *p<* 0.01). PAA was also associated with incident CKD (RR, 1.52; 95% CI, 1.11–2.08; FDR *p* = 0.02). The inverse PAA-CVD estimate (RR, 0.64; 95% CI, 0.43–0.95; FDR *p* = 0.04) was based on 29 events and was treated as exploratory rather than evidence of protection (**Table 7**).

**Table 7:** Associations of PAA and FI-Lab with individual incident diseases at 12 months.

Outcome	Exposure	*N*	Events	RR (95% CI)	*p*-value	FDR-adjusted *p*-value
Diabetes	PAA	916	28	1.77 (1.31–2.40)	< 0.01	< 0.01
	FI-Lab	916	28	1.66 (1.24–2.23)	< 0.01	< 0.01
CKD	PAA	969	34	1.52 (1.11–2.08)	< 0.01	0.02
	FI-Lab	969	34	1.26 (0.91–1.75)	0.17	0.20
CVD	PAA	943	29	0.64 (0.43–0.95)	0.02	0.04
	FI-Lab	943	29	1.10 (0.78–1.55)	0.58	0.58

Risk ratios are per 1-SD higher exposure and were adjusted for age, sex, and center. False-discovery rate-adjusted *p*-values were calculated across the six disease-specific tests.

### 3.5 Exploratory longitudinal changes at approximately 12 months

Repeated laboratory data were available for 820 participants (463 from Shandong and 357 from Jiangsu). After recalculating follow-up PAA with chronological age set to baseline age + 1 year, pooled mean PAA increased from − 0.90 (8.59) years at baseline to 2.00 (8.78) years at 12 months, a mean increase of 2.90 (3.06) years (*p<* 0.01). The mean PAA changes were 2.84 (3.29) years in Shandong and 2.97 (2.74) years in Jiangsu (both *p<* 0.01). FI-Lab increased from 0.08 (0.08) to 0.10 (0.09), a mean change of 0.02 (0.06) (*p<* 0.01). In inverse-probability-weighted analyses, the mean PAA change was 2.89 years [standard error (SE), 0.11; *p<* 0.01] and the mean FI-Lab change was 0.02 (SE, 0.00; *p<* 0.01), with an effective sample size of 794.86 (**Table 8**; **Figure 5**). In the longitudinal center-specific-residual sensitivity analysis, the pooled PAA change was 3.19 years (Shandong, 2.84 years; Jiangsu, 3.66 years; all *p<* 0.01), confirming the direction of change while showing that its magnitude depends on the age–Phenotypic Age reference relationship. These changes are presented as exploratory descriptive findings and should not be interpreted as an annualized rate of biological aging. Exact person-specific reassessment intervals were unavailable.

**Table 8:** Exploratory within-person changes in PAA and FI-Lab at approximately 12 months by center and in the pooled cohort.

Measure	Cohort	*N*/ESS	Baseline mean (SD)	12-month mean (SD)	Mean change (SD or SE)	*p*-value
PAA (years)	Shandong	463	− 0.48 (8.07)	2.36 (8.09)	2.84 (3.29)	< 0.01
FI-Lab	Shandong	463	0.10 (0.09)	0.11 (0.09)	0.01 (0.07)	< 0.01
PAA (years)	Jiangsu	357	− 1.44 (9.21)	1.53 (9.59)	2.97 (2.74)	< 0.01
FI-Lab	Jiangsu	357	0.06 (0.06)	0.08 (0.07)	0.02 (0.05)	< 0.01
PAA (years)	Pooled	820	− 0.90 (8.59)	2.00 (8.78)	2.90 (3.06)	< 0.01
FI-Lab	Pooled	820	0.08 (0.08)	0.10 (0.09)	0.02 (0.06)	< 0.01
PAA (years)	Pooled IPW	ESS 794.86	–	–	2.89 (0.11)	< 0.01
FI-Lab	Pooled IPW	ESS 794.86	–	–	0.02 (0.00)	< 0.01

Unweighted p-values are from paired *t*-tests. Stabilized inverse-probability-weighted estimates address selective availability of annual laboratory reassessment. Follow-up PAA was recalculated using baseline chronological age + 1 year in the fixed Shandong reference equation; a center-specific-residual sensitivity analysis is reported in the Results. This longitudinal analysis is exploratory; exact person-specific reassessment intervals were unavailable, and the reported PAA change should not be interpreted as an annualized biological-aging rate.

*ESS*, effective sample size.

**Figure 5:**
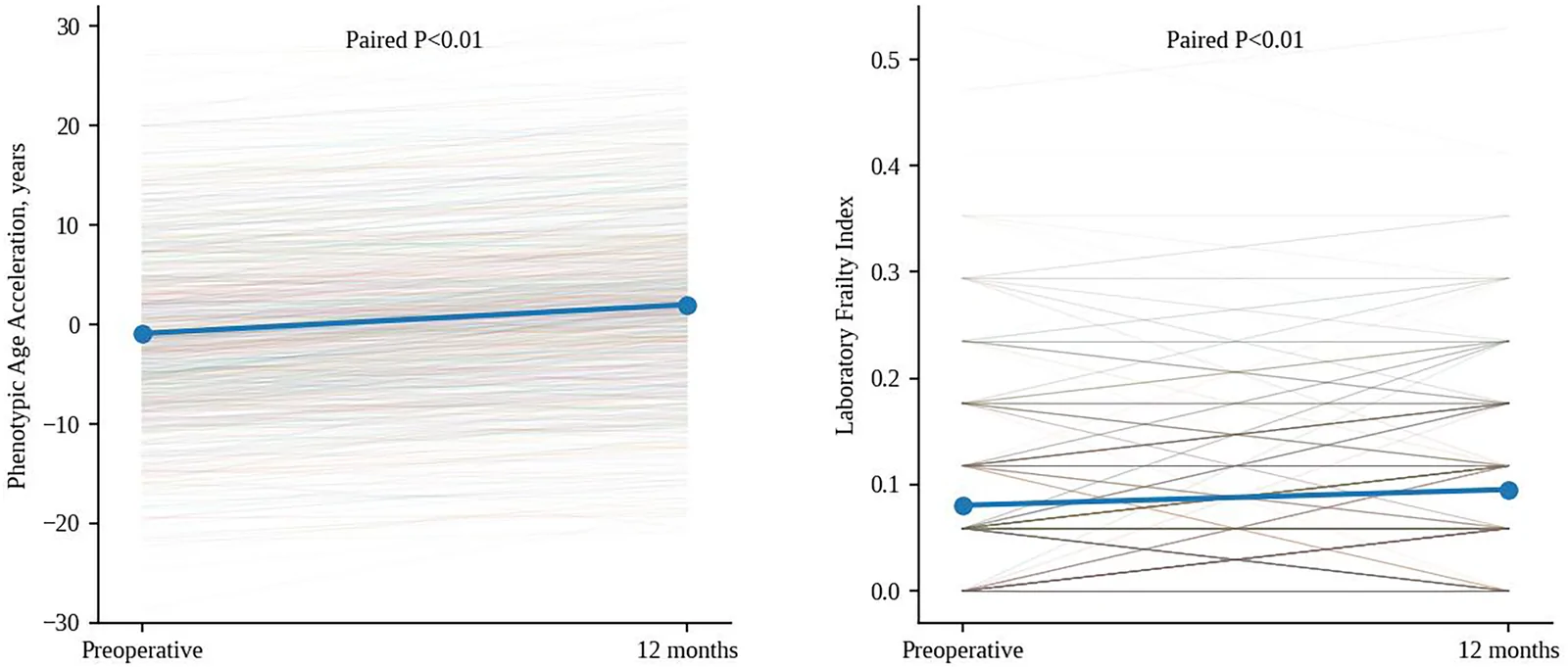
Exploratory within-person changes in PAA and FI-Lab from baseline to approximately 12 months in the pooled cohort. Participant-level trajectories are shown with pooled means. After follow-up, PAA was recalculated using baseline age + 1 year; PAA increased by 2.90 years and FI-Lab by 0.02 (both *p<* 0.01). These changes are descriptive.

### 3.6 Additional and sensitivity analyses

The baseline regression slope of Phenotypic Age on chronological age was 1.66 in Shandong (intercept, − 48.02) and 0.98 in Jiangsu (intercept, − 8.14). Consequently, under the fixed Shandong reference, PAA was essentially uncorrelated with chronological age in Shandong (r approximately 0.00) but strongly inversely correlated with age in Jiangsu (*r* = − 0.74; *p<* 0.01). Accordingly, the unadjusted PAA comparison in **Table 1** and the Kaplan–Meier categorization in **Figure 3** are presented as descriptive analyses; the age-adjusted regression models constitute the primary inferential analyses. This marked center difference in the age–Phenotypic Age relation is the reason the center-specific residual analysis is emphasized as the key sensitivity analysis for PAA.

PAA and FI-Lab were modestly correlated (Pearson *r* = 0.25; *p<* 0.01), and their center-specific distributions are shown in **Figure 6**. In age-, sex-, and center-adjusted fixed-horizon models, PAA was associated with mortality by 90, 180, and 365 days (ORs, 1.54, 1.71, and 1.60; *p* = 0.03, *p<* 0.01, and *p<* 0.01), while FI-Lab showed corresponding ORs of 1.48, 1.51, and 1.59 (*p* = 0.04, *p* = 0.01, and *p<* 0.01); neither index was significantly associated with 30-day mortality (PAA *p* = 0.39; FI-Lab *p* = 0.36) (**Figure 7**). Findings were robust when PAA was recalculated using center-specific age–Phenotypic Age residuals and exposures were standardized within center (PAA HR, 1.39; 95% CI, 1.09–1.77; *p<* 0.01; FI-Lab HR, 1.28; 95% CI, 1.04–1.57; *p* = 0.02) and to exclusion of deaths within 30 days (PAA HR, 1.47; 95% CI, 1.09–1.98; *p* = 0.01; FI-Lab HR, 1.35; 95% CI, 1.02–1.78; *p* = 0.04).

**Figure 6:**
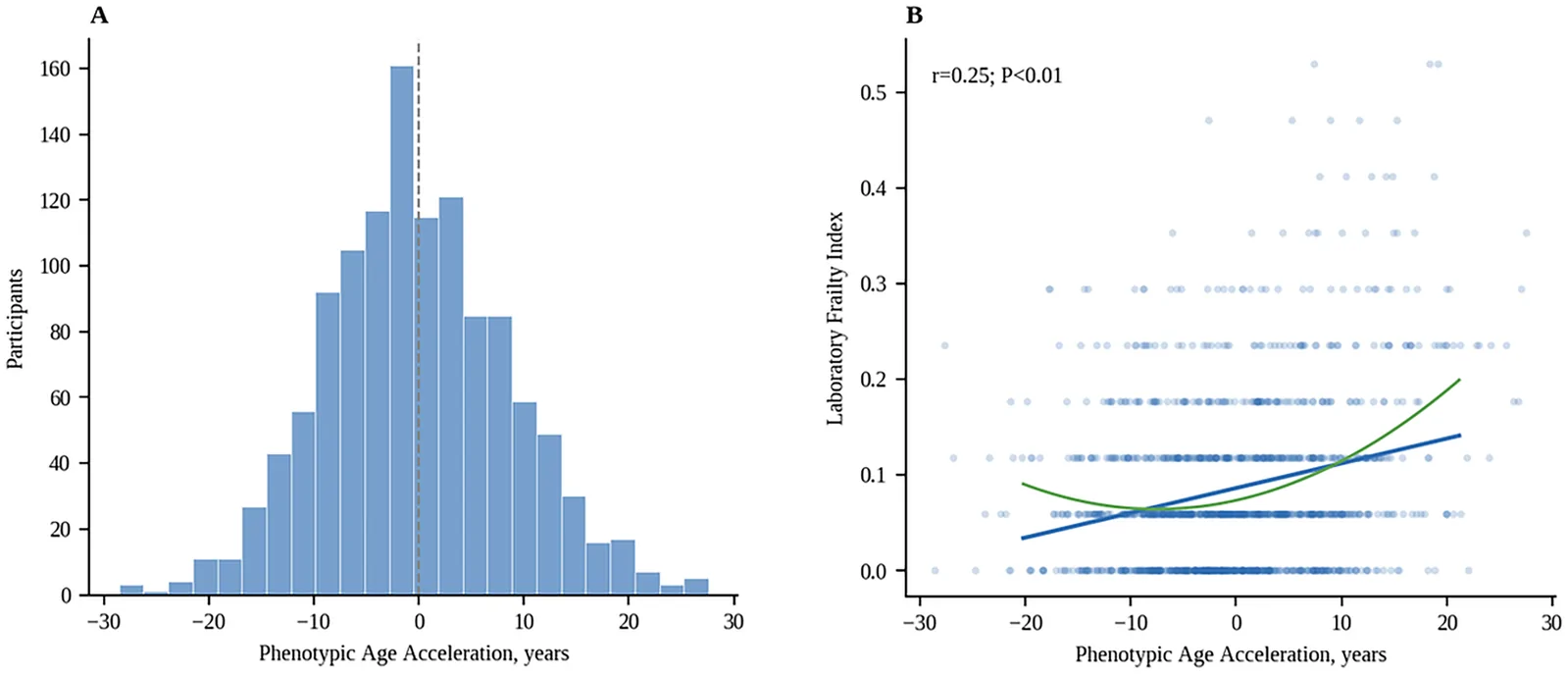
Baseline distributions and relationship between PAA and FI-Lab. **(A)** Center-specific PAA distributions. **(B)** Correlation between PAA and FI-Lab in the pooled cohort (Pearson *r* = 0.25; *p<* 0.01).

**Figure 7:**
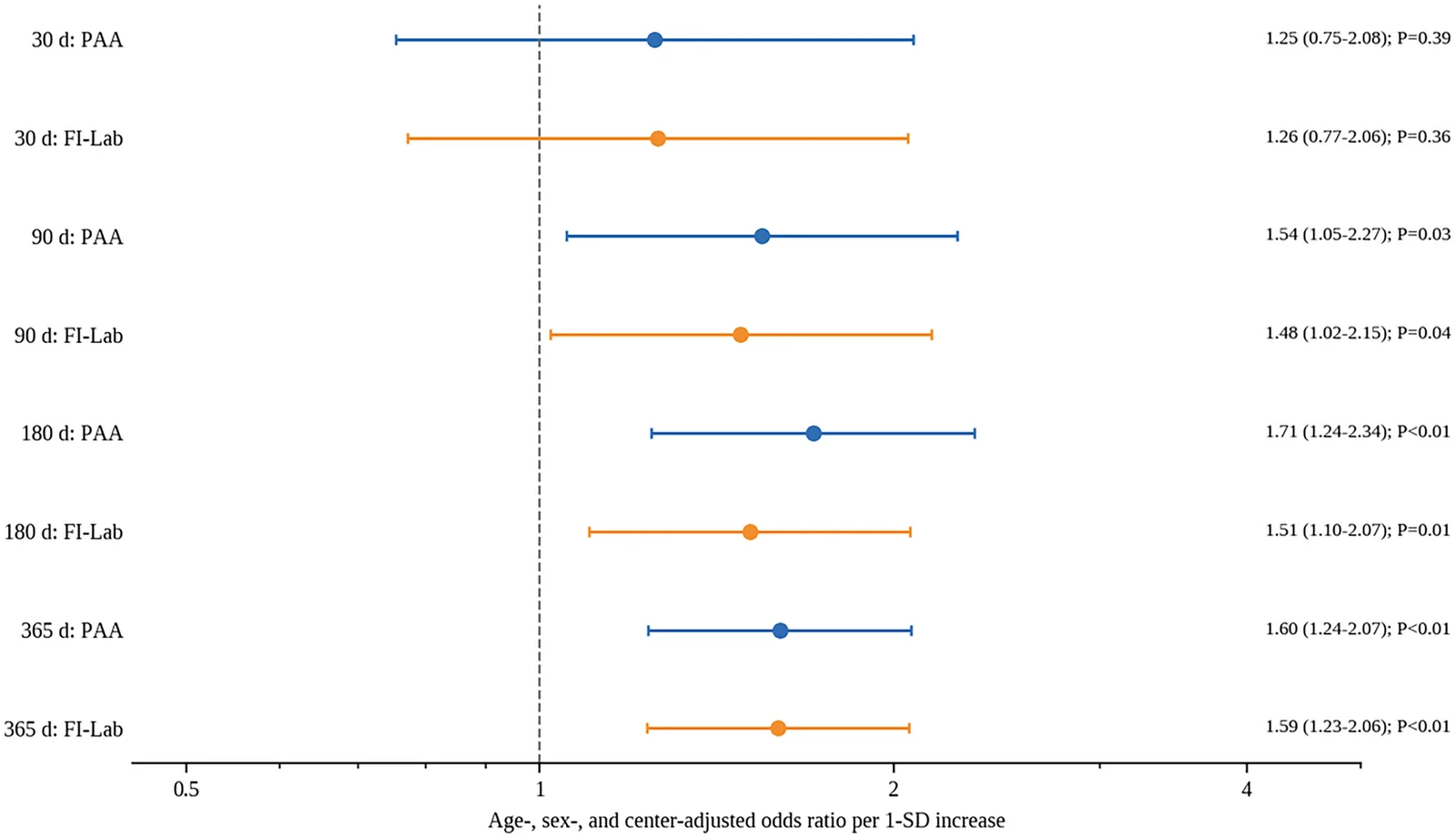
Age-, sex-, and center-adjusted associations of PAA and FI-Lab with mortality at fixed postoperative horizons. Points indicate odds ratios per 1-SD increase, horizontal lines indicate 95% CIs, and *p*-values are shown for each estimate.

## 4 Discussion

Higher PAA and FI-Lab were associated with postoperative mortality in the minimally adjusted models. After full adjustment in the center-stratified analysis, the association remained for PAA, whereas the FI-Lab estimate was borderline. The PAA center-interaction estimate was close to the null but imprecise (interaction ratio, 1.06; 95% CI, 0.68–1.65), while the FI-Lab estimate suggested possible heterogeneity (1.48; 95% CI, 0.94–2.32). The prediction analyses were less favorable: neither marker produced a clear increase in discrimination over the study clinical model, and decision-curve analysis showed no consistent gain in net benefit. An association that was reproducible across cohorts therefore did not necessarily provide reliable incremental prediction in a new center. Importantly, the primary external-validation AUC of the study clinical model was only 0.60 (95% CI, 0.45–0.73); therefore, the lack of incremental improvement should be interpreted relative to this study-specific comparator and not as evidence that the biomarkers cannot add value beyond a stronger or differently specified clinical prediction model.

The PAA results are consistent with a growing perioperative literature on biologic aging. Phenotypic Age combines biomarkers from several organ systems in a mortality-derived score intended to reflect physiologic dysregulation beyond chronological age (10, 11). Our mortality estimates were directionally consistent with those of the international surgical study by Scarpa et al. (12), and with multicenter reports relating accelerated biologic aging to postoperative acute kidney injury (13) and intraoperative hypotension (14). In the fixed-horizon analyses, associations were present at 90, 180, and 365 days but not at 30 days, when only 14 deaths had occurred. This timing is compatible with PAA reflecting broader physiologic reserve and recovery, although the limited number of early deaths prevents a firm comparison across horizons.

The age dependence of the fixed-reference PAA is an important methodological caveat. The age–Phenotypic Age slope differed substantially between centers (1.66 in Shandong vs. 0.98 in Jiangsu), so applying the Shandong relation without re-estimation induced a strong inverse correlation between PAA and age in Jiangsu. For this reason, the unadjusted between-center PAA summary and Kaplan–Meier categories should be interpreted descriptively rather than as age-independent effects. The primary mortality models explicitly adjusted for age, and the fully adjusted association remained similar when PAA was redefined as a center-specific age–Phenotypic Age residual before center-specific standardization (HR, 1.39; 95% CI, 1.09–1.77; *p<* 0.01). Nevertheless, absolute PAA values derived from a fixed reference should not be assumed to be directly interchangeable across institutions with different age–Phenotypic Age relations. We therefore limit our transportability claim to the robustness and direction of the age-adjusted association under alternative residual definitions; we do not claim that the absolute fixed-reference PAA scale itself is transportable or interchangeable between the two centers.

FI-Lab measures vulnerability by counting accumulated laboratory abnormalities rather than applying a mortality-derived weighting scheme (15, 16). Higher values have been linked to mortality and other adverse outcomes in community studies (17, 18) and to clinically important postoperative outcomes in surgical cohorts (19, 20). In our data, FI-Lab was also associated with the 12-month composite of death or incident cardiometabolic-renal disease, including in the model that contained PAA. Its components span renal, metabolic, inflammatory, nutritional, hepatic, hematologic, and electrolyte domains, several of which may become abnormal before a clinical diagnosis is recorded. At the same time, the stronger mortality association in Jiangsu and the interaction *p*-value of 0.09 indicate that a common FI-Lab scale may not behave identically across institutions. Laboratory measurement units were harmonized, both hospitals used routine internal quality-control procedures, and local reference intervals were broadly similar. However, analyzer manufacturer/model and reagent-level metadata were unavailable, so assay- or calibration-related differences cannot be excluded as a contributor to the higher FI-Lab distribution in Shandong or to center-specific effect estimates.

The direction of abnormality is important. The conventional FI-Lab assigns the same deficit count to values below and above the prespecified interval, even though their pathophysiologic implications may differ. We retained this established deficit-accumulation coding to preserve construct comparability and to avoid developing a new, data-driven laboratory risk score in a dataset with only 74 deaths. A direction- or magnitude-sensitive laboratory score may be informative, but it would require separate development and validation in a substantially larger cohort.

Adjustment for FRAIL, ASA physical status, and comorbidity attenuated both laboratory-derived measures. That attenuation is expected to some extent because these variables capture overlapping aspects of physiologic vulnerability. Questionnaire-defined frailty and deficit accumulation are related but not interchangeable constructs (6, 7); CCI reflects chronic disease burden (28), and ASA physical status summarizes perioperative disease severity (29, 30). Overlap also exists between PAA and FI-Lab themselves: nine of the 17 FI-Lab components are used in the Phenotypic Age equation. Their modest correlation and the loss of statistical significance when both were entered together suggest shared information rather than two fully independent biomarker domains.

External validation clarified the gap between risk association and deployable prediction. Model performance can change when case mix and baseline risk differ between development and validation samples (21). Calibration is particularly sensitive to such shifts and therefore requires direct assessment in external data (22, 36). In Shandong-to-Jiangsu validation, FI-Lab improved NRI and IDI but did not significantly improve AUC, Brier score, or decision-curve net benefit; PAA produced smaller changes. In the reverse validation, discrimination rose numerically after laboratory aging measures were added, but calibration slopes for the extended models were below 1. Given that reclassification statistics can appear favorable without corresponding gains in discrimination, calibration, or net benefit (24), they are better interpreted alongside these other measures (23). On the basis of these data, PAA and FI-Lab function as risk markers, but their value as portable additions to perioperative prediction remains unproven. In the primary validation, calibration-slope point estimates of 2.61, 4.38, and 2.01 for the study clinical model, study clinical model + PAA, and combined models suggest that predicted risks may have been too compressed, although the very wide confidence intervals all included 1. These point estimates therefore reinforce, rather than reduce, the need for caution about calibration in a cohort with only 23 deaths within 365 days. Accordingly, our interpretation prioritizes discrimination, calibration, Brier score, and decision-curve net benefit; NRI and IDI are considered supplementary and do not alter the overall conclusion when these core performance measures do not improve consistently. The modest discrimination of the study clinical model also means that conclusions about incremental value are specific to this comparator.

The reverse Jiangsu-to-Shandong exercise should be interpreted more cautiously than the primary validation: although the prediction model was trained in Jiangsu, the PAA reference equation and standardization parameters were derived from Shandong. We therefore regard this direction as a secondary transportability sensitivity analysis rather than an independent external validation.

The 12-month analyses broadened the outcome beyond mortality. Diabetes, CKD, and CVD often coexist within the cardiovascular-kidney-metabolic continuum (31, 32), and cardiovascular-kidney-metabolic risk stages are common in adults (39). Both laboratory-derived measures were associated with incident diabetes, while PAA was also associated with incident CKD after multiplicity correction. The inverse association between PAA and incident CVD should be viewed cautiously: only 29 CVD events occurred, and the estimate is inconsistent with the wider biologic-aging literature. Residual confounding, competing risks, ascertainment, and chance are all plausible explanations. Studies outside surgical settings have linked Phenotypic Age to distinct aging phenotypes and later morbidity (40, 41). A single composite aging measure may therefore relate differently to different organ-specific outcomes.

In the exploratory longitudinal analysis, PAA and FI-Lab increased at the approximately 12-month reassessment. After recalculating follow-up PAA using baseline age +1 year, the pooled mean PAA change was 2.90 years; a center-specific-residual sensitivity analysis gave a pooled change of 3.19 years. These values should not be interpreted as indicating 2.90 or 3.19 calendar years of biological aging over a single year. PAA is a residual measure whose longitudinal change reflects both biomarker change and the selected age–Phenotypic Age reference relation. The FI-Lab increase remained 0.02. These longitudinal changes are descriptive rather than evidence of a surgical effect. Without a nonsurgical comparison group, the study cannot separate postoperative change from calendar aging, intercurrent illness, treatment, regression to the mean, or selective return for repeat testing. The consistency of the direction across centers does, however, show that repeated measurement is feasible. Future work should use prospectively recorded exact reassessment intervals and externally transportable age-reference functions to determine whether persistent elevation or failure to return toward baseline is more informative than a single preoperative value. In addition, exact participant-specific reassessment intervals were unavailable, which further precludes interpreting the observed PAA change as a precisely annualized biological-aging rate.

Several design features strengthen the analysis: laboratory measurement units were harmonized across centers, FI-Lab used a prespecified 17-item definition, laboratory measures were compared with questionnaire-defined frailty, and both center-specific and center-stratified association models were used. Reciprocal external validation also kept development and validation samples separate. Prediction performance was examined with discrimination, PR-AUC, Brier score, calibration, reclassification, and decision-curve methods rather than a single metric. The main limitation is the number of events. Only 74 deaths occurred by 540 days, and the primary external-validation cohort had 23 deaths within 365 days, which limited precision for calibration, interaction, and incremental-performance estimates (25, 27). The health-examination-based cohorts may not represent the broader surgical population. We also lacked cause-specific mortality, standardized postoperative complications, readmission, and length of stay; annual disease outcomes were not formally adjudicated; laboratory cutoffs may not capture all between-center assay differences; repeat testing was selective despite weighting; and residual confounding remains possible. Larger multicenter cohorts are needed before these measures are used for individual clinical decisions. In addition, the fixed Shandong age–Phenotypic Age reference did not reproduce the same age slope in Jiangsu, making age-reference transportability a specific limitation of absolute PAA comparisons despite the age-adjusted and center-specific-residual sensitivity analyses. Individual-level baseline data for excluded candidates were unavailable, so we could not formally compare included with excluded participants; given that the analytic cohorts represented only 19.0% and 16.2% of the initial candidate pools, selection bias related to complete-data eligibility remains possible. Although laboratory units were harmonized and local reference intervals were broadly similar under routine internal quality control, the absence of analyzer/reagent metadata means platform-related differences cannot be excluded. Exact person-specific reassessment intervals were also unavailable for the exploratory longitudinal analysis.

Accordingly, the absence of statistically significant calibration-intercept or calibration-slope tests in the primary external-validation cohort should not be read as evidence of good calibration; with only 23 deaths within 365 days, calibration and incremental-performance estimates remain highly uncertain. In addition, individual procedure names were not modeled as separate covariates; operative heterogeneity was represented by the prespecified surgical-stress grade and cancer-related-surgery indicator, so residual procedure-specific differences in baseline risk may remain. Thus, the width of the confidence intervals, rather than nonsignificant hypothesis tests, should be the primary basis for judging the uncertainty of calibration and incremental-performance estimates.

## 5 Conclusion

Preoperative PAA and FI-Lab were associated with postoperative vulnerability in two hospital cohorts, with PAA showing the more consistent mortality association after full adjustment and a similar association after center-specific residualization. However, the fixed Shandong PAA scale was not age-independent in Jiangsu and should not be considered directly transportable as an absolute measure. Neither marker consistently improved externally validated prediction beyond the study clinical model, whose discrimination was modest; calibration also remained imprecise because of the limited number of validation events. Routine perioperative use should therefore await validation in larger, more diverse multicenter datasets with better assay-level harmonization and evidence of added clinical net benefit.

## Data Availability

The original contributions presented in the study are included in the article/supplementary material. Further inquiries can be directed to the corresponding author.
